# Consumers’ Attitudes toward the Use of an Edible Coating for Lamb Meat According to Label Information

**DOI:** 10.3390/foods11030323

**Published:** 2022-01-24

**Authors:** Raquel Alves Mauricio, Rosires Deliza, Renata Tieko Nassu

**Affiliations:** 1 Faculdade de Ciências Farmacêuticas de Araraquara, Universidade Estadual Paulista (FCFAr/UNESP), Rodovia Araraquara Jaú, Km 01-s/n-Campos Ville CEP, Araraquara 14800-903, SP, Brazil; raquel.a.mauricio@unesp.br; 2 Embrapa Agroindústria de Alimentos, Avenida das Américas, 29501, Rio de Janeiro 23020-470, RJ, Brazil; rosires.deliza@embrapa.br; 3 Embrapa Pecuária Sudeste, Rodovia Washington Luiz, Km 234, Fazenda Canchim, São Carlos 13560-970, SP, Brazil

**Keywords:** chitosan, conjoint analysis, packaging, purchase intention

## Abstract

The use of edible coatings in meat is currently being investigated in several studies. However, consumers’ attitudes toward this technology are not clear. This study evaluated consumers’ intention to purchase vacuum-packaged meat with a chitosan coating based on the label information. Three factors combined with different levels were considered: type of cut (“loin”, “French rack”, or “leg steak”), coating/information (no information, chitosan without information explained, and chitosan with information explained), and price (low or high). Consumers’ purchase intentions were evaluated using a 7-point structured scale (1 = certainly would not buy; 7 = certainly would buy). The data were analyzed by conjoint analysis and cluster analysis. The average purchase intention value was 4.3 ± 0.5. The “French rack” cut showed the highest value for purchase intention and a high price was also a relevant factor. The packaging with the label stating the presence of a “chitosan” coating without giving any further information obtained higher purchase intentions than the one with the more detailed information label. In the cluster analysis, the factor “type of cut” showed the highest relative importance in two groups, while “price” had a higher impact in another. The type of cut was the main factor affecting consumers’ purchase intentions. However, different attitudes were observed depending on the group.

## 1. Introduction

There are currently 18.9 million sheep in in Brazil [1], mainly in the northeastern and southern regions. The average consumption of lamb meat across the world is 1.7 kg per capita [2]; however, this figure is much lower (0.6 kg per year) in Brazil [3], and restricted in specific areas of the country. Despite its low consumption, lamb meat has a high level of acceptance and is associated with special occasions and sharing with other people (e.g., barbecues, friends, and parties) [4].

Appearance plays an essential role in consumers’ purchase intentions and meat acceptance and is especially important for beef and lamb meat [5]. Thus, strategies have to be developed to achieve improvements in this intrinsic factor of the product. The use of edible coatings, combined with vacuum packaging, is one option for the improvement of this parameter, as oxidation can change the color of the product. In addition to appearance, other characteristics of a meat product, such as origin, price, and processing information, are also related to its perceived quality and may affect consumers’ choices [6]. Information on labels is especially relevant in innovative products, as consumers do not have previous knowledge of the product [7,8]. Moreover, issues related to sustainability, nutritional quality, and processing presented on the label affect consumers’ purchase intentions [9]. Labels offer direct communication between the industry and consumers and can positively influence their attitudes toward food when they are familiar with the information presented [10]. Personal characteristics affect the time spent on analyzing labels and consumers’ perceptions of them [11]. Studies on consumers’ perceptions of labels show that consumers lack comprehension about relevant terminologies and concepts [12,13]. Conjoint analysis can be used to study the effect of a group of factors related to products—such as their brand, price, packaging color, and label—and their impact on consumers’ purchase intentions. This analysis allows the estimation of the contribution of each factor and level and their relative importance to consumers’ preferences or intentions to purchase a product. Therefore, conjoint analysis is useful for creating and evaluating new products designed to meet the needs of consumers [14].

Red meat is usually sold refrigerated or frozen in Brazil, and consumers are not used to or aware of innovations and new technologies applied to meat conservation and processing. Therefore, consumers can be resistant to new meat-based products, justifying the need to evaluate the best strategy with which to communicate new technologies being applied to products in order to reduce the risk perception of consumers [15]. In a situation where they perceive risk, consumers tend to avoid purchasing a food rather than running the risk of purchasing or consuming it [16,17]. Many studies have reported the effect of the use of chitosan films and coatings on meat quality, such as those reviewed by Sanchez-Ortega et al. [18]. There are no specific Brazilian regulations for the use of edible coatings and films for foods in general. These are considered to be ingredients or additives, following Brazilian government regulations and the considerations of the Codex Alimentarius from Food and Drugs Administration (FDA) guidelines. However, coatings could potentially be used for extending the shelf life of products, and there is a lack of information regarding consumers’ perceptions of the use of edible coatings on lamb meat. This study investigated the effect of label information on consumers’ intention to purchase vacuum-packaged lamb meat with an edible coating.

## 2. Materials and Methods

The Ethical Committee approved this experiment for research in human beings under protocol CAEE 16295919.6.0000.5504. The participants completed an informed consent form at the beginning of the online study.

### 2.1. Participants

This study comprised 230 regular and potential consumers of lamb meat. These data were obtained through non-probabilistic convenience sampling, considering only the participants’ availability, as described by Deliza et al. (2019) [19].

A total of 60 participants out of 230 answered they did not or would never consume the product or gave the same score for all treatments. Therefore, they were excluded, leaving the remaining 170 participants. The majority of the participants were female (63.5%). Of the total participants, 26.5% were between 36 and 45 years, and 29.4% were between 46 and 55 years, 75.9% had undergraduate education, 60.6% lived in the southeastern region of Brazil, and 36.5% had an income between 10 and 20 minimum wages. The participants reported an occasional consumption of lamb meat (49.4%) and acknowledged that they read the information on the product label frequently (35.3%) or always (35.3%) (Table 1).

### 2.2. Experimental Design and Label Preparation

Three factors, combined with different levels, were studied in a rating-based conjoint analysis. The study design investigated the type of cut (loin, French rack and leg steak), the coating (no information, with information (1) “with chitosan edible coating, technology for preserving meat properties”, and with information (2) “with chitosan edible coating”, and price (low and high). No additional information about the properties of edible packaging (biodegradability, non-toxicity, and antibacterial or antioxidant properties) or about the packaging processing methods used (e.g., extrusion, dipping, spraying, panning, or fluidization) were provided to the participants. The factors and levels were chosen according to the criteria described in the following. The types of cut were chosen according to the variety of products found in the retail market: French rack, also known as “carré”, which is bone-in loin corresponding to the Longissimus lumborum muscle with eight last thoracic ribs, which is one of the most popular cuts used for culinary preparations; boneless loin, which is the same muscle as the previous one; and the leg steak (a unique cut provided by the studied brand, featuring a leg cut in a steak). Regarding the price, it was determined according to the average minimum and maximum prices of these cuts at retail businesses. The “coating” factor was defined considering information on how labels detailing genetically modified ingredients, nanotechnology, irradiation, and organic foods affect consumer behavior [20,21].

The combination of all factors/levels generated 18 treatments, which meant there were too many stimuli to be evaluated, which would fatigue the participants. Therefore, we used a fractioned factorial design and nine treatments (Table 2) to investigate the effects of the three variables on consumers’ intention to purchase vacuum-packed lamb meat.

**Figure 1:**
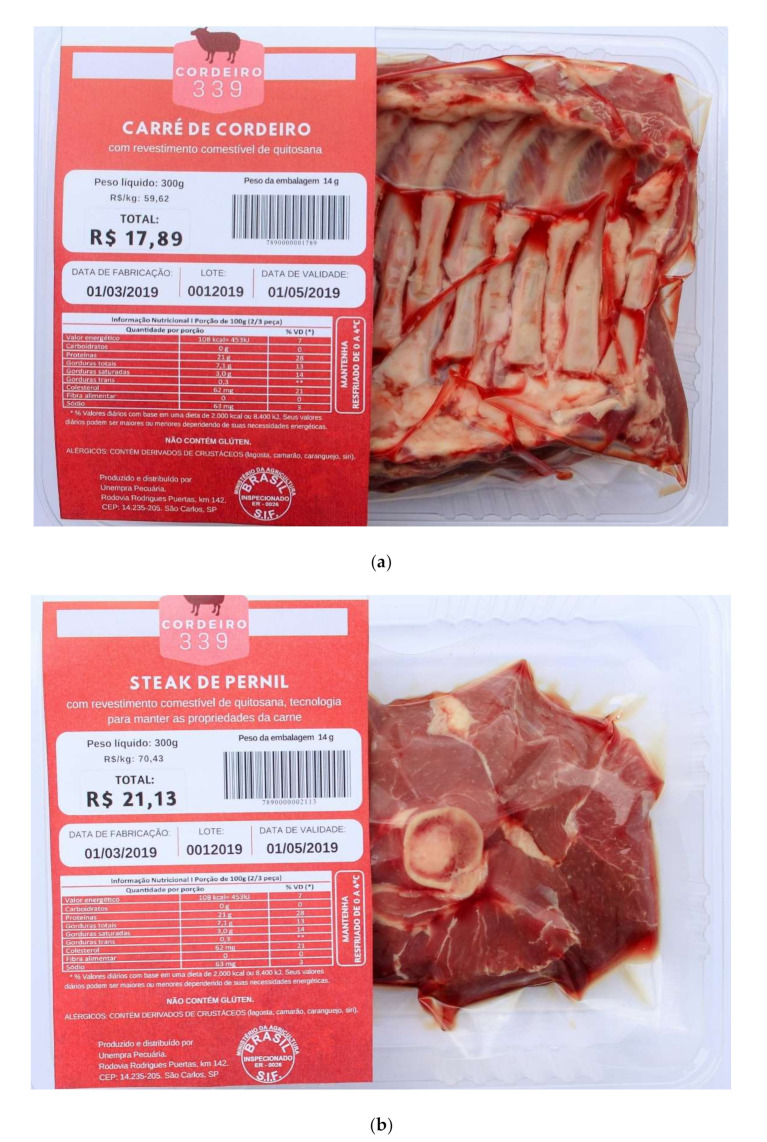
Examples of labels and packagings used in the study (**a**) treatment 8: chitosan/information, French rack cut (“carré”), low price and (**b**) treatment 9: chitosan/explained information, leg steak cut, high price.

The preparation of the stimulus followed the Brazilian regulations for food and beverage labeling according to ANVISA [22,23,24,25]. The labels were created containing the information listed in Table 1 using a fictitious brand name. The designs of the labels were inspired by labels on vacuum-packaged meat available on the retail market. The CANVA software was used (http://www.canva.com, version Canva Pty Ltd., Sydney, Australia) (accessed on 21 February 2019). These labels were placed in terephthalate polyethylene trays containing the lamb meat cuts. Images of the packaging were taken under natural daylight using a professional photographic camera (Canon Inc., model EOS Rebel T5i, Tokyo, Japan). Different lamb meat cuts were obtained at specialized butcheries and a 300 g sample was used in each packaging. Two examples of the stimuli used in the study are shown in Figure 1.

### 2.3. Experimental Procedures

The study was carried out using an on-line questionnaire developed as Google Docs (https://www.google.com/intl/pt-BR/forms/about/, accessed on 19 May 2019), which contained the stimuli as pictures of packaged lamb meat with the label containing information on the type of cut, price, and chitosan coating, according to the experimental design shown in Table 1. The link to the study was presented to consumers using social media. The consumer purchase intent was evaluated with a 7-point structured scale (1 = certainly would not buy; 4 = perhaps would or not buy; 7 = certainly would buy) [26,27].

In the questionnaire, the first question concerned the habits of participants regarding lamb meat consumption. In the event the consumer reported never having eaten or that they would never eat lamb meat, only social-demographic answers were presented. However, if the consumer answered that they had consumed or had an interest in consuming the product, they would be directed to the pictures to respond the questionnaire regarding their purchase intention using the given scale. After finishing the rating-based conjoint task, participants were asked to answer questions related to the social-demographic characteristics, which included the region of the country where they lived as well as questions about their attitude towards label and nutritional information using a 5-point Likert scale (1 = totally disagree; 5 = totally agree”).

### 2.4. Data Analyses

#### 2.4.1. Data on Purchase Intention

Data on the purchase intention and social-demographic characteristics were analyzed by the descriptive analysis. From the purchase intention scores, an agglomertive hierarchical cluster analysis was applied and three clusters of consumers with similar utilites for the conjoint variables (coating/information, type of cut, and price) were identified. Euclidean distances and Ward’s aggregation criterion were considered. The Kruskal–Wallis test was applied to compare the clusters. The software version 2019.1.1 (Addinsoft^®^, New York, NY, USA) was used for the statistical analyses.

#### 2.4.2. Conjoint Analysis

In the conjoint analysis, the relative importance was calculated and the effect of each factor on the purchase intention and the utilities, which measure how consumers assess the product by a combination of each factor [28]. An agglomerative hierarchical cluster analysis considering Euclidean distances and using Ward’s method was performed to obtain the segmentation of consumers. Afterward, conjoint analysis was applied again, but only on the clusters.

## 3. Results

### 3.1. Purchase Intention and Factors of Labels Affecting It

The average purchase intention of consumers was between “perhaps buying or not buying” and “would probably buy” (4.3; SD = 0.5). A significant difference (*p* < 0.05) was found between the average values of purchase intention for the clusters. The highest value was found in cluster 1 (5.3; SD = 0.4) when compared to the values obtained for cluster 2 (3.1; SD = 0.5) and cluster 3 (3.6; SD = 1.1)

The Likert scale shows a group of affirmatives that reflect the attitude related to the studied subject and indicates the degree of agreement of consumers [29]. In our results, the expiration date (84.7% agree or totally agree) and price (74.7% agree or totally agree) were the factors that most influenced the consumers’ purchase intentions. The factors “information about ingredients”, “nutritional facts”, and “information about additives” significantly affected (*p* < 0.05) the purchase intention, adding the category responses “agree” and “totally agree” (64.2%, 60.0%, and 61.8%, respectively). The product brand was considered less important than the other factors (36.5% agree and 14.7% totally agree). Among the three different clusters used in this study, there was no difference between the consumers’ opinions related to the factors mentioned above.

### 3.2. Social-Demographic Data

Regarding socio-demographic data, significant associations between age x education (χ² = 73.553; *p* < 0.001), age x income (χ² = 86.539; *p* < 0.001), and education x income (χ² = 42.326; *p* < 0.001) were observed After the cluster analysis, three groups of consumers with similar responses were identified; however, the social-demographic characteristics did not show significant differences (*p* > 0.05) among them.

### 3.3. Conjoint Analysis

**Figure 2:**
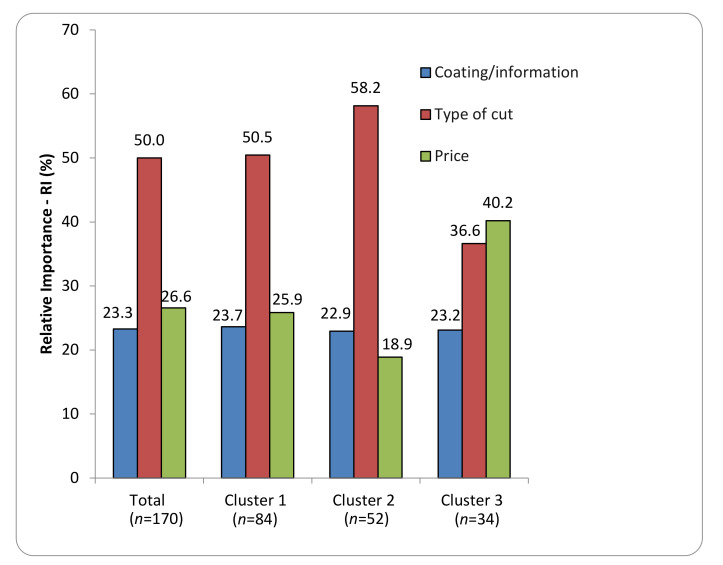
Relative importance (RI) of different factors for total consumers and clusters.

In the conjoint analysis, a statistic model for multiple regression analysis (Minim, 2013) estimated the contributions of factors/levels as well as their relative importance (RI). The RI represents the impact of each factor on the consumer purchase intention (Minim, 2013). The RI shows how consumers evaluate the value of a product by combining each contribution level. Figure 2 shows the RI of each factor for all consumers and clusters.

Regarding RI (Figure 2), “type of cut” was the most relevant at the aggregate level (RI = 50.0%), followed by price (RI = 26.6%) and coating/information (RI = 23.3%) in the group of total consumers as well as for clusters 1 and 2 (RI = 50.5% and 58.2%, respectively, Figure 2). However, this factor was not so relevant for cluster 3. The “coating/information” factor was similar for the total group and clusters. “Price” played an important role in cluster 3, with an RI = 40.2%, differently from the general group and cluster 1. The cluster 3 had a minor importance.

Regarding the part-worth utilities, for the “type of cut” factor the “French rack” (carré) showed the highest value (0.093), implying its positive contribution to the purchase intention at the same aggregate level as the high-priced product (Table 3). On the other hand, “chitosan” with information explained (“technology for preservation of meat”) had a negative value for utilities (−0.101). The type of cut “leg steak” had the highest value for participants in cluster 1 and 2 (0.122 and 0.342, respectively), but the opposite occurred in Cluster 3, which had a utilities value of −0.817 and “loin” had the highest value (0.448) (Table 3). However, the “high” level for price positively contributed to the aggregate and other clusters. Regarding the coating/information factor, the level “chitosan, explained information” contributed negatively to the purchase intention in all clusters. Particularly for cluster 2, in addition to this level the level “No information” had a negative value (−0.088). Consumers in cluster 2 valued the “chitosan without explained information” which positively contributed to consumers’ intention to buy lamb meat (Table 3).

## 4. Discussion

The highest utility value was achieved by the “loin with bone” (0.093) at the aggregate level, suggesting that it could improve the consumer purchase intention, as it is the most commercialized lamb cut in Brazil and very popular in culinary preparations. Although “loin” is the same muscle deboned, it showed the lowest value for utilities (−0.095), as Brazilian consumers are not familiar with this specific lamb meat cut. These results demonstrate the relationship between familiarity with the product and the purchase intention. Previous studies have reported that consumers evaluate the information available on the package/label before they make the decision to purchase a product. This experience with the product affects their confidence and, consequently, their purchase intention [16,17,30]. The result that high-priced lamb cuts were relevant to the consumer purchase intention, with positive utilities for “price” in all clusters, could be explained by the fact that lamb meat is less often consumed than chicken, beef, or pork. Only 10.6% of the participants in this study frequently eat lamb meat, and it is known that lamb meat is eaten only on special occasions. Thus, apparently, there is a willingness to pay more for it. De Andrade et al. (2017) [31] reported consumers’ intention to purchase lamb meat in different contexts, where the occasion had the biggest effect on the purchasing intention, as this meat is mainly eaten on a special date rather than at typical meals.

Socio-demographic characteristics affect consumers’ attitudes toward labels and, consequently, their intention to purchase food. In this study, a significant proportion of consumers (76.5%) had an income higher than five minimum wages. Considering that label observation, purchase intention, and willingness to pay are affected by factors such as education and income, “high price” presented a positive utility in all clusters. This shows that these variables may be affected by the socio-characteristics of the population studied. Although “price” has already been reported as one of the factors less likely to affect consumers’ intention to purchase lamb meat [32], high price is considered to be a quality characteristic of a product. However, if income is not consistent with the product price, the purchase intention will be reduced [33].

As expected, a relation between an increase in age and education was observed. The higher education of the participants used in this study was associated with a higher income. Most consumers (93%) in this study had undergraduate education (17.0%) or a graduate degree (75.9%). Higher education is generally positively associated with a higher income. Therefore, besides the positive effect of higher price on the purchase intention of these products, the frequency of reading information on labels could be justified. In this study, 70.6% of the participants acknowledged that they always (35.3%) and frequently (35.3%) read information on labels. The reading frequency of labels has been reported in other studies to be proportional to a higher education level [34,35]. Consumers with higher education usually read labels and use them to acquire information about products more frequently than consumers with lower education levels do [36]. Therefore, information on labels should be clear to all consumers.

Regarding the coating/information factor, samples with the information “chitosan edible coating” without an explanation on the label showed a positive effect. In contrast, when there was an explanation about chitosan (“technology for preserving meat properties”), a negative utility value was observed. Information without a detailed description did not affect the purchase intention. Consumers seemed to be satisfied when informed about the product, affecting their acceptance and purchase intention [8]. However, the presence of more information on the label can negatively influence consumers, also affecting their perception [37]. In this study, information on chitosan seemed to evoke a rejection of the product, as chitosan may have been associated with the use of additives and consumers tend to relate these to non-natural products, which affects their purchase intention [38]. This result also suggests that consumers are concerned about the presence of additives and tend to avoid products containing these types of compounds. Additives are perceived as risky, without long-term benefits, and their effects are unclear, causing uncertainty to consumers [39].

Frequently, the information available is used to reduce risks during the purchasing process, such as attention to the product shelf life, where 84.7% of participants agreed that information on shelf life is important when purchasing any food.

This study suggested that associating the benefit of new technology with the product, providing information on the product to consumers, and avoiding an association with the use of additives are useful strategies. Moreover, it is important for consumers to familiarize themselves with the intrinsic aspects of the product by offering a tasty new product, contributing to consumers’ purchase intention [40].

## 5. Conclusions

Consumers’ intention to purchase vacuum-packaged lamb meat with an edible coating was affected by factors other than the technology applied. A detailed explanation of the use of chitosan contributed negatively to the product image, affecting the consumers’ purchase intentions. Therefore, marketing strategies to inform the consumer about the advantages of using edible coatings are needed to boost consumers’ confidence. The high price of the lamb meat at retailers in Brazil contributed positively to increasing the consumers’ purchase intentions and it may have been associated with high-quality lamb meat. However, due to the profiles of the participants (high income and education), further studies including consumers with different profiles are needed. These results contribute to the development of marketing strategies that inform consumers of the advantages of the use of edible coatings made with chitosan.

## Figures and Tables

**Table 1:** Frequency of socio-demographic characteristics of participants.

	Frequency (%)			
Socio-Demographic Parameters	Total (*n* = 170)	Cluster 1 (*n* = 84)	Cluster 2 (*n* = 52)	Cluster 3 (*n* = 34)
Gender				
Male	36.5	32.1	48.1	35.3
Female	63.5	67.9	51.9	64.7
Age				
18 to 25 years	8.8	4.8	17.3	5.9
26 to 35 years	17.6	17.9	21.2	8.8
36 to 45 years	26.5	33.3	17.3	23.5
46 to 55 years	29.4	25.0	30.8	41.2
56 to 65 years	10.6	11.9	7.7	11.8
More than 65 years	7.1	7.1	5.8	8.8
Education				
Elementary/middle school	-	-	-	-
High school	5.3	3.6	7.7	5.9
Technical school	1.8	1.2	1.9	2.9
Undergraduate	17.0	11.9	25.0	20.6
Graduate	75.9	83.3	65.4	70.6
Region				
North	0.6	-	-	2.9
Northeast	13.5	11.9	17.3	8.8
Central-West	15.3	19.0	5.8	20.6
Southeast	60.6	58.3	67.3	61.8
South	10.0	10.7	9.6	5.9
Income				
Less than 2 minimum wages *	4.7	2.4	9.6	2.9
3 to 5 minimum wages	18.8	13.1	26.9	17.6
5 to 10 minimum wages	15.9	20.2	9.6	17.6
10 to 20 minimum wages	36.5	41.7	32.7	29.4
More than 20 minimum wages	24.1	22.6	21.2	32.4
Lamb meat consumption				
Never	18.2	21.4	21.2	17.6
Occasionally	49.4	51.2	46.2	38.2
Sometimes	21.2	15.5	25.0	29.4
Frequently	10.6	10.7	7.7	14.7
Always	0.6	1.2	-	-
Label reading				
Never	3.5	2.4	3.8	5.9
Occasionally	10.0	9.5	9.6	11.8
Sometimes	15.9	11.9	13.5	26.5
Frequently	35.3	35.7	38.5	29.4
Always	35.3	40.5	34.6	26.5

* minimum wage = R$ 998.00 (US$ 255.00) in January 2019.

**Table 2:** Factors and levels of the fractionated factorial design used in this study.

Treatment n.	Coating/Information	Type of Cut	Price
1	No information	French rack (carré)	High R$ 32.20 (US$ 8.21)
2	Chitosan, explained information ^1^	French rack (carré)	High R$ 32.20 (US$ 8.21)
3	Chitosan, explained information ^1^	Loin	Low R$ 18.36 (US$ 4.68)
4	Chitosan, information ^2^	Loin	High R$ 39.57 (US$ 10.09)
5	No information	Leg steak	Low R$ 12.03 (US$ 3.06)
6	Chitosan, information ^2^	Leg steak	High R$ 21.13 (US$ 5.39)
7	No information	Loin	High R$ 39.57 (US$ 10.09)
8	Chitosan, information ^2^	French rack (carré)	Low R$ 17.89 (US$ 4.56)
9	Chitosan, explained information ^1^	Leg steak	High R$ 21.13 (US$ 5.39)

^1^ “With chitosan edible coating, technology for preserving meat properties”. ^2^ “With chitosan edible coating”.

**Table 3:** Part-worth utilities of each investigated factor and levels for the intent to purchase of coated lamb meat.

		Utilities			
Factor	Levels	Total (*n* = 170)	Cluster 1 (*n* = 84)	Cluster 2 (*n* = 52)	Cluster 3 (*n* = 34)
Coating/information	Chitosan, information ^2^	0.084	0.007	0.130	0.203
	Chitosan, explained information ^1^	−0.101	−0.037	−0.043	−0.346
	No information	0.084	0.030	−0.088	0.144
Type of cut	French rack (“carre”)	0.093	−0.144	0.297	0.369
	Loin	−0.095	0.022	−0.639	0.448
	Leg steak	0.001	0.122	0.342	−0.817
Price	High	0.440	0.358	0.263	0.914
	Low	−0.440	−0.358	−0.263	−0.914

^1^ With chitosan edible coating, technology for preserving meat properties; ^2^ With chitosan edible coating.

## Data Availability

The data presented in this study are available in the article.
